# Factors influencing psychological well-being in patients with Parkinson’s disease

**DOI:** 10.1371/journal.pone.0189682

**Published:** 2017-12-15

**Authors:** Alessandra Nicoletti, Giovanni Mostile, Fabrizio Stocchi, Giovanni Abbruzzese, Roberto Ceravolo, Pietro Cortelli, Marco D’Amelio, Maria F. De Pandis, Giovanni Fabbrini, Claudio Pacchetti, Gianni Pezzoli, Alessandro Tessitore, Margherita Canesi, Mario Zappia

**Affiliations:** 1 Department “G.F. Ingrassia”, Section of Neurosciences, University of Catania, Catania, Italy; 2 Institute of Neurology, IRCCS San Raffaele Pisana, Rome, Italy; 3 Centre for Parkinson’s Disease and Movement Disorders, DINOGMI, University of Genoa, Genoa, Italy; 4 Department of Clinical and Experimental Medicine, University of Pisa, Pisa, Italy; 5 IRCCS Institute of Neurological Sciences of Bologna, Bologna, Italy; 6 DIBINEM, Alma Mater Studiorum, University of Bologna, Bologna, Italy; 7 Department of Experimental Biomedicine and Clinical Neurosciences, University of Palermo, Palermo, Italy; 8 Department of Neurorehabilitation, Parkinson Operative Unit, San Raffaele Institute, Cassino (FR), Italy; 9 Department of Neurology and Psychiatry, and IRCSS Neuromed, La Sapienza University of Rome, Rome, Italy; 10 Parkinson’s Disease and Movement Disorders Unit, IRCCS Neurological National Institute C. Mondino, Pavia, Italy; 11 Parkinson Institute, ASST G. Pini—CTO, Milan, Italy; 12 Department of Medical, Surgical, Neurological, Metabolic and Aging Sciences, Second University of Naples, Naples, Italy; Instituto Cajal-CSIC, SPAIN

## Abstract

**Background:**

Both motor and non-motor symptoms could contribute to significant deterioration of psychological well-being in patients with Parkinson's disease (PD). However, its assessment has been only indirectly evaluated using tools based on health-related quality of life (HRQoL), such as the PDQ-39 scale.

**Objectives:**

To evaluate psychological well-being in PD using a specific tool of assessment, the Psychological Well-being Scale (PWS), and its clinical correlates.

**Methods:**

This article reports data of patients’ perception of health state, as measured by means of the PWS, from an epidemiological, cross-sectional study conducted in Italian PD patients (FORTE Study). We tested possible relationship between well-being and clinical characteristics including fatigue, depression, sleep disruption and HRQoL.

**Results:**

272 patients completed the PWS questionnaire. Significant and clinically-relevant correlations were found between PWS total score and Parkinson’s Fatigue Scale, Beck Depression Inventory, UPDRS Section I, PD Sleep Scale and PDQ-39 for HRQoL scores. Only clinically negligible correlations were found between PWS and motor scores.

**Conclusions:**

Non-motor symptoms have a significant impact on psychological well-being in PD patients.

## Introduction

Parkinson's disease (PD) is a progressive disorder associated with a wide range of motor and non-motor symptoms that contribute to significant disability and deterioration of health-related quality of life (HRQoL) [1,2].

In 1948 the World Health Organization's (WHO) defined “health” in terms of “physical, mental, and social well-being, and not merely the absence of disease and infirmity” [3]. The term “subjective well-being” or “happiness” is often used to refer to a combination of the absence of negative emotions and the presence of positive emotions, life satisfaction, and social engagement [4]. While HRQoL represents a broad concept which includes the physical state of health as well as social-economic factors, in accordance with the concept of “health”, well-being is particularly related with the psychological dimension, as reinforced by the definition of “mental health” proposed for the WHO [5,6].

“Well-being” is now commonly proposed as a theme for outcome measures as it reflects the expanded goals of treatment, from medical treatment toward broader health care. Several reliable ways of measuring well-being are available, including measures that focus on the presence of positive emotions and the absence of negative emotions, life satisfaction [7], social engagement [8], and physical wellness [9]. Such measures of subjective well-being emphasize the importance of the hedonic aspects of experience, such as pleasure, satisfaction, and happiness [10].

Treatment of PD has traditionally focused on the improvement of motor symptoms. More recently, treatment has widened to include also non-motor symptoms. Even if the measurement of quality of life using specific scale, such as the PDQ-39 [11], has become a common endpoint in almost all the clinical trials as well as observational studies recently carried out, few studies have systematically investigated the well-being in terms of positive emotions and life satisfaction. There is still a need for specific indicators to be used in clinical trials targeting on health state perception in PD, which differ from the other common indicators of quality of life. This is crucial for quantifying possible specific therapeutic interventions on psychological well-being in PD.

The objective of the present study is to evaluate psychological well-being and positive functioning in a large sample of PD patients from an observational, cross-sectional, multicentre study carried out in Italy (i.e. the FORTE study) [12], in order to identify factors possibly related to a better “well being”.

## Methods

The FORTE study plan included a cross-sectional single visit during which all information was collected. The study population included adult outpatients of either sex with idiopathic PD diagnosed according to the U.K PD Society brain bank diagnostic criteria for PD [13], including new diagnosed patients. Exclusion criteria included the presence of any type of dementia (DSM-IV criteria) [12]. The demographic and general characteristics of study participants have been previously reported [12]. Briefly, the study was conducted in 27 sites in Italy between March and June 2011. A total of 402 patients were screened and all were eligible for inclusion into the study (245 men, 60.9%; age 66.9±8.9 years; disease duration 7.5±5.6 years).

The following data were recorded at the study visit: patient demographics, medical history (onset and duration of PD), presence of co-morbidities and associated treatments, severity of PD according to the modified Hoehn-Yahr scale. Psychological well-being was evaluated using the psychological well being scales (PWS), a standardized non-disease specific instrument [14,15], already validated in Italian language [16]. PWS is a 84 item self-rating inventory, measuring six multiple facets of psychological well-being (14 items for each dimension): “autonomy”, “environmental mastery”, “personal growth”, “positive relations”, “purpose in life” and “self-acceptance”. Patients reported their degree of agreement ranging from 1 (disagree) to 6 (fully agree), with negative items counted inversely to obtain a final score for each domain, in which higher scores are indicative of better outcome.

The degree of fatigue was evaluated using the 16-items Parkinson’s Fatigue Scale (PFS) [17], a patient-rated scale exploring physical aspects of fatigue and its impact on daily functioning, which is based on 16 items ranging from 1 (strongly disagree) to 5 (strongly agree), with the PFS-16 mean score being calculated as the mean of all individual item scores [12,17]. Other measures used in the study included the Unified Parkinson’s Disease Rating Scale (UPDRS) for motor assessment [18], the Parkinson's disease questionnaire-39 item version (PDQ-39) for quality of life [19], the Beck Depression Inventory (BDI) [20] for depression and the Parkinson’s Disease Sleep Scale (PDSS) for sleep disorders [21]. All evaluations were performed together with the UPDRS, which was administered during the”ON” phase.

The study protocol was approved by the Ethics Committee of the coordinating centre (Comitato Etico IRCCS San Raffaele Pisana, Rome) and each of the participating sites. All patients provided written informed consent prior to any study-related procedure was started.

### Statistical analysis

The comparison between groups for continuous parameters was performed by means of Mann-Whitney test. The analysis of variance (ANOVA) was also used to compare more than two independent mean groups for quantitative parameters. The pairwise comparisons were done applying the Scheffe’s method when the p-value associated to the F-test of the ANOVA was statistically significant.

A multiple linear regression analysis model was also used to assess the relationship between PWS total score and the following variables: age, age at diagnosis for PD, duration of disease, Hoehn-Yahr stage, PDSS total score, BDI total score, PFS-16 mean score, PDQ-39 total score, UPDRS total score and the subtotal scores for the Sections I-IV. In this model, a backward procedure with a cut-off of p = 0.10 was applied to select the variables to be removed. Pearson correlation coefficients (r) were computed to estimate the linear relationship between the PWS total score and the above variables. As role of thumb, correlation coefficients between -0.3 and 0.3 were considered clinically negligible [22]. The statistical testing was conducted at the two-sided α = 0.05 level.

## Results

Overall 272 PD patients completed the PWS questionnaire. The age was 66.2±9.4 years (range 37–89) and approximately 65% of patients were females. The duration of the disease was 7.3±5.8 years (range 1–40).

The mean (±SD) PWS total score in the overall evaluable population was 348.7±46.2 (median 350, range 222–454). Table 1 shows the results of PWS as total score and in each dimension of health in the overall evaluable population. The level of impairment was comparable in each domain, with mean values ranging from 55.7 for personal growth to 60.2 for autonomy.

**Table 1:** Results of PWS total and single domains scores.

**PWS total score**	348.72 ± 46.19 (222–454)
**Autonomy**	60.18 ± 8.60 (35–84)
**Environmental mastery**	56.98 ± 9.51 (32–83)
**Personal growth**	55.74 ± 8.22 (28–80)
**Positive relations**	60.03 ± 10.21 (26–83)
**Purpose in life**	57.33 ± 9.56 (28–78)
**Self-acceptance**	58.46 ± 10.65 (19–82)

**Notes:** Data are mean ± SD (range).

Distribution of the PWS score by gender, age class, educational level, stage of the disease and current treatment for PD are showed in Table 2. The difference between genders (p = 0.358) and education level groups (p = 0.473) were not statistically significant. The mean PWS total score in patients aged 70–74 years was significantly higher than that of those aged ≥75 years, without statistically significant differences between the other age ranges, as well as in the comparison between patients aged ≤70 years (351.22±45.03) and those aged >70 years (344.82±48.71) (p = 0.370). Patients with stage 1 of the Hoehn-Yahr scale showed a higher mean PWS total score than the other stages of the scale (significantly vs. stage 2 and pooled stage 3–4). Although patients treated with monoamine oxidase inhibitors (MAOIs) had a higher mean PWS total score than the other drug classes, the difference by category of current treatments for PD was not statistically significant (p = 0.133).

**Table 2:** PWS total score by demographic and baseline clinical characteristics.

	No. of patients	Mean ± SD	Median	Range (min-max)
**Gender**				
Males	94	351.4 ± 46.6	352.50	226–454
Females	178	344.6 ± 45.8	348.50	222–435
**Age class (years)** *				
<60	57	358.4 ± 47.5	364.0	222–454
60–64	52	347.8 ± 46.5	349.5	226–446
65–69	55	340.3 ± 40.3	340.0	235–435
70–74	61	362.1 ± 44.9	366.0	238–434
≥75	47	332.2 ± 47.5	334.0	245–448
**Education Level**				
None/First level	77	355.5 ± 44.0	356.0	239–435
Second Level	80	343.9 ± 52.3	347.5	222–454
High school	83	348.5 ± 42.0	348.0	226–445
University	32	347.6 ± 47.1	347.0	245–448
**Hoehn & Yahr scale** **				
Stage 1	71	364.9 ± 48.1	375.0	256–454
Stage 2	129	342.7 ± 43.8	344.0	226–446
Stage 3	66	346.8 ± 44.8	348.5	222–435
Stage 4	6	320.0 ± 56.3	326.0	254–385
**Current treatment for PD** †				
MAOIs	107	357.7 ± 45.3	359.0	226–454
Dopamine agonist	182	352.8 ± 44.3	355.0	222–454
Levodopa	207	345.3 ± 46.4	345.0	222–448
Amantadine	11	346.6 ± 40.0	342.0	285–391
Anticholinergic drugs	7	332.7 ± 44.5	322.0	277–399

Notes

*p value (F-value test) = 0.004. Difference between age 70–74 and ≥ 75 years: 29.96 (95% CI: 2.66 to 57.26); NS in the other pairwise comparisons.

**p value (F-value test) = 0.004. Difference between Stage 1 and Stage 2: -22.20 (95% CI: -35.42 to -8.98); difference between Stage 1 and Stage 3–4: 31.54 (95% CI: 9.70 to 53.37); NS in the other pairwise comparisons.

† A patient may be counted in more than one drug category.

Results of the correlation tests are summarized in Table 3. A direct correlation was found between PWS total score and PDSS total score (r = 0.349, p<0.0001), while a moderate inverse correlation was found between PWS total score and the following variables: PFS total score (r = -0.398, p<0.0001); BDI total score (r = -0.569, p<0.0001); PDQ-39 total score (r = -0.544, p<0.0001) and UPDRS Section I (r = -0.488, p<0.0001). Less evident correlations were found between PWS total score and the other UPDRS sections, as well as with the severity of the disease (Hoehn-Yahr scale), whereas PWS total score resulted to be not related with and age at diagnosis of PD and duration of disease. The “environmental mastery” was the PWS dimension of health most strongly correlated with the severity of non-motor symptoms, showing also a moderate inverse correlation with UPDRS total and UPDRS Section II scores.

**Table 3:** Results of correlation tests.

	PWS total	Autonomy	Environmental mastery	Personal growth	Positive relations	Purpose in life	Self acceptance
**Duration of PD**	r: -0.104 p = 0.087	r: -0.036 p = 0.561	r: -0.128 p = 0.035	r: -0.061 p = 0.321	r: -0.084 p = 0.167	r: -0.130 p = 0.033	r: -0.064 p = 0.291
**Age at diagnosis**	r: -0.024 p = 0.693	r: -0.021 p = 0.729	r: 0.044 p = 0.474	r: -0.188 p = 0.002	r: 0.003 p = 0.961	r: -0.064 p = 0.291	r: 0.074 p = 0.226
**Hoehn & Yahr**	r: -0.177 p = 0.003	r: -0.081 p = 0.183	r: -0.200 p = 0.001	r: -0.153 p = 0.012	r: -0.122 p = 0.045	r: -0.177 p = 0.003	r: -0.131 p = 0.031
**PFS total**	**r: -0.398 p<0.001**	r: -0.236 p<0.001	**r: -0.437 p<0.001**	r: -0.261 p<0.001	r: -0.241 p<0.001	**r: -0.442 p<0.001**	**r: -0.314 p<0.001**
**BDI total**	**r: -0.569 p<0.001**	**r: -0.364 p<0.001**	**r: -0.564 p<0.001**	**r: -0.357 p<0.001**	**r: -0.403 p<0.001**	**r: -0.555 p<0.001**	**r: -0.511 p<0.001**
**UPDRS total**	r: -0.262 p<0.001	r: -0.143 p = 0.021	**r: -0.306 p<0.001**	r: -0.143 p = 0.021	r: -0.073 p = 0.005	r: -0.299 p<0.001	r: -0.205 p = 0.001
**UPDRS Section 1**	**r: -0.488 p<0.001**	r: -0.212 p = 0.001	**r: -0.487 p<0.001**	**r: -0.351 p<0.001**	**r: -0.355 p<0.001**	**r: -0.510 p<0.001**	**r: -0.444 p<0.001**
**UPDRS Section 2**	r: -0.241 p<0.001	r: -0.117 p = 0.058	**r: -0.320 p<0.001**	r: -0.103 p = 0.095	r: -0.151 p = 0.014	r: -0.270 p<0.001	r: -0.199 p = 0.001
**UPDRS Section 3**	r: -0.176 p = 0.004	r: -0.113 p = 0.063	r: -0.194 p = 0.001	r: -0.097 p = 0.111	r: -0.115 p = 0.060	r: -0.214 p = 0.000	r: -0.121 p = 0.048
**UPDRS Section 4**	r: -0.153 p = 0.013	r: -0.040 p = 0.518	r: -0.215 p = 0.000	r: -0.062 p = 0.315	r: -0.087 p = 0.159	r: -0.186 p = 0.002	r: -0.140 p = 0.022
**PDSS total**	**r: 0.349 p<0.001**	r: 0.184 p = 0.003	**r: 0.372 p<0.001**	r: 0.219 p = 0.001	r: 0.286 p<0.001	**r: 0.306 p<0.001**	**r: 0.310 p<0.001**
**PDQ total**	**r: -0.544 p<0.001**	**r: -0.330 p<0.001**	**r: -0.539 p<0.001**	**r: -0.330 p<0.001**	**r: -0.413 p<0.001**	**r: -0.514 p<0.001**	**r: -0.488 p<0.001**

**Notes:** data are Pearson’s correlation coefficients (r) and p values. Pearson’s r indicative of an at least moderate inverse or direct correlation (i.e. ≤ -0.3 or ≥ 0.3) are highlighted in bold.

Selected predictive variables (age, age at diagnosis for PD, duration of disease, Hoehn-Yahr stage, PDSS total score, BDI total score, PFS-16 mean score, PDQ-39 total score, UPDRS total and subtotal scores for the Sections I-IV) were then included in the regression analysis setting PWS total score as dependent variable. After ten step of backward elimination the following variables were kept in the regression model: BDI total score (coefficient: -1.725, S.E. 0.418, p<0.0001), PDQ-39 total score (coefficient: -0.928, S.E. 0.231, p<0.0001) and the UPDRS section I (coefficient: -4.841, S.E. 1.625, p = 0.0032) (ANOVA F = 36.46, p<0.0001; goodness of fit: R^2^ = 0.3794).

## Discussion

The main findings of our analysis have shown a mean PWS score indicative of a moderate impairment of psychological well-being and positive functioning in the cohort of PD patients that took part in the cross-sectional Italian FORTE study [12] and completed the PWS questionnaire. The level of well-being was comparable across the six dimensions of health, in line with the high correlation coefficients between each of them reported by both the developers of the scale [15] and in the Italian validation of PWS [16].

PWS total and domains mean scores were shown also to be not correlated with age, disease duration and motor symptoms. Conversely, the mean score of PWS overall and in each dimension of health resulted to be inversely correlated with the severity of fatigue and depression, and was directly correlated with the extent of sleep disruption. Overall, these findings were indicative of a worsened psychological well-being in patients with higher impairments of health state. As further evidence of the influence of the mood and cognitive components on the psychological perception of well-being, important correlations were found between almost all PWS dimensions (except for autonomy) and the “Mentation, Behavior, and Mood” section score of the UPDRS. The “environmental mastery”, that is defined as the ability to manage complex environments to suit personal needs and values, was the PWS dimension of health most strongly correlated with the severity of non-motor symptoms.

The significant effect of BDI total score, PDQ-39 total score and the UPDRS section I in the regression analysis model allowed building a model indicative of a moderate predictive effect of the exploratory variables. The results of the stepwise multiple linear regression analysis were comparable to those reported in other studies, in which UPDRS Section I (but not UPDRS motor Section III) were kept in the model [23,24].

Overall, these findings provide a confirmatory evidence of previous data showing that neuropsychiatric symptoms, especially depression, night-time sleep disorders, fatigue and somnolence, are the variables that most affect the HRQoL of patients with PD [24–26]. Thus, our data demonstrate that non-motor symptoms are negatively associated with patients’ health perception. Notably, the mean PWS total score and subscores were found to correlate with the mean total score of the PDQ-39, which is the most used and validated disease-specific instrument for self-reported health status in PD [27,28]. However, it should be noted that, differently from PDQ-39, no clinically-relevant correlation between PWS and motor scores was detected, being variables also excluded by the multiple linear regression model. As indicator of the overall concept of health, in fact, PDQ-39 score has shown to be dependent on patients motor status, correlating with Hoehn-Yahr staging and total UPDRS motor score [21]. Psychological well-being, and thus the patient’s health state perception, seems instead to be more dependent on non-motor symptoms referred by patients with PD, including fatigue, depression and sleep disruption. Nevertheless, some of these aspects, such as fatigue, could be due to other physical factors related to motor symptoms, thus we cannot exclude motor components to be also associated to psychological well-being in PD.

The cross-sectional design of the study may represent a principal limitation. Another study limitation may be due to the inclusion of patients mainly in the early stages of the disease, that may have contributed to the weak correlation between PWS total score and domains and the Hoehn-Yahr score. Moreover, since about 38% of the variability of the PWS total score can be accounted for by the obtained linear regression model, we cannot exclude that other not-investigated factors may have contributed to PWS total score variability in the study population.

In conclusion, to the best of our knowledge this is the first study which evaluates psychological well-being instead of common measures of QoL as indicator of health status in PD. The results of our data suggest that the presence of non-motor symptoms, such as fatigue, depression and sleep disruption, have a significant impact on psychological well-being in PD patients. Therefore, our findings confirm the previous evidence on the burden of non-motor symptoms as a key determinant of psychological well-being in PD. Future studies are needed to evaluate possible pharmacological interventions on psychological well-being in PD.
